# Prevalence and causes of anemia among older adults in India: findings from wave 2 of the Harmonized Diagnostic Assessment of Dementia for the Longitudinal Aging Study in India (LASI-DAD)

**DOI:** 10.1186/s12939-025-02671-4

**Published:** 2025-11-14

**Authors:** Anushikha Dhankhar, Pranali Khobragade, Geeta Chopra, Joyita Banerjee, Sandy Chien, Sarah Petrosyan, Masroor Anwar, Shweta Sharma, A. B. Dey, Jinkook Lee, Eileen Crimmins, Peifeng Hu, Sharmistha Dey, Bharat Thyagarajan

**Affiliations:** 1 Center for Economic and Social Research, University of Southern California, Los Angeles, CA USA; 2 Metropolis Healthcare Ltd., Delhi, India; 3 Department of Biophysics, All India Institute of Medical Sciences, Delhi, India; 4 Department of Laboratory Medicine and Pathology, Division of Molecular Pathology and Genomics, University of Minnesota Twin Cities, Minneapolis, MN USA; 5 Department of Epidemiology, School of Public Health, University of Minnesota, Twin Cities, Minneapolis, MN USA; 6 Venu Geriatric Institute, Venu Eye Hospital and Research Centre, Delhi, India; 7 Department of Economics, University of Southern California, Los Angeles, CA USA; 8 Davis School of Gerontology, University of Southern California, Los Angeles, CA USA; 9 Division of Geriatrics, David Geffen School of Medicine, University of California Los Angeles, Los Angeles, CA USA; 10 Advanced Research Diagnostics Laboratory, MMC 512 1200 Washington Ave. S, Minneapolis, MN 55415 USA

**Keywords:** Anemia, Iron deficiency anemia, Nutritional anemia, Anemia of chronic disease, Anemia of chronic kidney disease, Undefined anemia

## Abstract

Anemia among older adults aged ≥ 60 years is a well-described risk factor that increases the risk of falls, cardiovascular diseases, and mortality. In India, objectively measured national estimates of anemia prevalence and the causes of anemia among older adults are lacking. The Harmonized Diagnostic Assessment of Dementia for the Longitudinal Aging Study in India (LASI-DAD) collected venous blood samples from a nationally representative sample of 3,252 individuals in wave 2 of the study. Out of these, 3,009 samples were used to estimate national prevalence and regional differences in anemia prevalence and its underlying causes. Anemia was defined as hemoglobin < 13 mg/dl in males and < 12 mg/dl in females and further categorized into nutritional and non-nutritional anemia based on several nutritional (ferritin, Vitamin B12, and folate), inflammatory (ferritin, C-reactive protein), and renal (serum creatinine) biomarkers. The overall national anemia prevalence was 49.92%. Anemia prevalence was significantly higher among women (53.9%) than men (45.8%). Among those with anemia, nutritional anemia was found in 63.5% of the respondents, with isolated iron deficiency anemia being the most common cause (51.8%). Among the non-nutritional category, anemia of chronic disease was the most common type (31.4%). About 10% of all anemia cases could not be classified into either category using the available data. Regional differences were seen with the highest prevalence of anemia in Assam, West Bengal, Jharkhand, and Odisha, and the lowest prevalence (< 30%) in Jammu & Kashmir and Haryana. Multivariate adjustment showed that age, sex, and region of residence are independently associated with anemia status. The study provides valuable insights into the overall anemia prevalence among older adults in India, its underlying causes, and regional differences to lay a strong foundation for making informed decisions toward anemia control in India.

**Supplementary Information:**

The online version contains supplementary material available at 10.1186/s12939-025-02671-4.

## Background

Anemia is recognized as a public health concern with a global prevalence of 24.3% across all age groups [1]. According to the Global Burden of Disease, Injuries, and Risk Factors study, the sub-Saharan African and South-Asian countries have the highest levels [1]. Children, adolescents, women of reproductive age, and older adults (≥ 60 years) have been identified to be at high risk of developing anemia [2]. A recent systematic review and meta-analysis of cross-sectional studies reported a pooled anemia prevalence of 68.3% among older adults in India [3]. Anemia can significantly impact an individual’s daily activities, especially older adults, as it adversely affects physical [4] and cognitive functioning [5, 6], increases the risk of falls [7], frailty [8], cardiovascular [9] diseases, and mortality [10].

The pathophysiology of anemia can either be multifactorial or result from a single cause [11]. In India, nutritional deficiencies [12] related to iron, vitamin B12, and folate are the predominant causes of anemia. The non-nutritional causes include the presence of chronic disease, such as chronic kidney disease [13], myelosuppression, inflammatory or autoimmune diseases, blood loss due to parasitic infections, and malignancies [2]. In the older population, in addition to the reasons mentioned above, low energy intake, nutrient malabsorption, anorexia, immunodeficiency, medications, and lower socioeconomic status, especially in India, play an important role in anemia development [14]. However, up to 30% of anemia cases among older adults may be unexplained and difficult to categorize [13].

### Statement of the problem

Several previous studies have attempted to estimate the prevalence of anemia and identify its causes among older adults in India. However, the evidence is limited as it reflects a small non-representative sample population mostly recruited at local institutions [15, 16] or hospitals [17, 18], in urban [19–24] or rural [25–32] community settings, but limited to one state [33–35]. Moreover, the prevalence of anemia reported in these studies varies widely, ranging from 21% to 96%.

In India, preventive measures to curb anemia were first implemented in 1970 through the National Anemia Prophylaxis Program [36]. However, since then, efforts have primarily been directed toward reducing anemia prevalence among children and women of reproductive age. In 2016, the Longitudinal Aging Study in India [37] (LASI) was launched as part of the National Program for the Health Care of the Elderly to study aging patterns in a nationally representative sample in India. The Harmonized Diagnostic Assessment of Dementia for LASI (LASI-DAD) studied a nationally representative sub-sample of LASI and collected venous blood samples. In this study, we report comprehensive national estimates of anemia prevalence and causes of anemia among older adults aged 60 years and above in India. Published literature indicates that regional variation in dietary, socio-economic, lifestyle, and cultural patterns may affect the distribution of different anemia subtypes and also help identify geographical clustering [38–40]. Therefore, we also present regional differences in anemia prevalence in the LASI-DAD sample.

## Methods

### Participants

The Longitudinal Aging Study in India (LASI) is an ongoing study that collects socioeconomic and health-related data from over 73,000 adults aged 45 years and above and their spouses. Its sample was drawn from the 2011 Census, representing each state and union territory as well as the country as a whole. A sub-sample of LASI was drawn to collect data from 4,096 to 4,638 LASI respondents during Wave 1 and Wave 2 of the LASI-DAD study, respectively [41, 42]. Wave 1 of the LASI-DAD study was launched in 2017, and wave 2 in 2022 to study late-life cognition and estimate the prevalence of dementia among older adults aged 60 years and above in India [41, 42]. Key demographic variables, such as age, gender, and urbanicity, were obtained from the main LASI and used in both waves of the LASI-DAD study [41–43]. Information on the highest level of education was obtained from the respondents during the interview and recorded as less than primary education, primary education, middle school, secondary education, higher secondary school, diploma/certificate holders, graduate degree, postgraduate degree, and professional degree. The detailed study design and methodology have been published elsewhere [41, 42]. The study was conducted according to the Declaration of Helsinki. Ethics approval was obtained from the Institute Ethics Committee of All India Institute of Medical Sciences (AIIMS), New Delhi, all collaborating medical institutions, and the University of Southern California (UP-15-00684). The Health Ministry Screening Committee of the Indian Council of Medical Research provided administrative clearance to the LASI-DAD research study (2202–16741/F1).

### Venous blood sample collection and processing

For Wave 2 of the LASI-DAD study, 3252 out of 4638 (70.1%) participants consented to the collection of a 17 mL venous blood specimen. Blood draws were conducted across India from November 2022 to April 2024. A written informed consent form was obtained from each respondent in the presence of a family member. A consent form was translated into the participant’s local language, and consent was obtained with the participant’s signature or a thumbprint in cases where the respondent could not read or write. For cognitively impaired participants, consent was obtained from a legal representative authorized to sign on their behalf. The samples were collected by trained phlebotomists at each respondent’s house and shipped under ambient temperature (2–8° Celsius) to the local Metropolis Healthcare Ltd. laboratory for processing within 4–6 h of collection. Detailed procedures for training of phlebotomists and other study staff have been previously described elsewhere [44]. The serum and plasma tubes were centrifuged at the local Metropolis Healthcare Ltd. laboratory before shipment to the central laboratory. Within 24 h of collection, these samples were then shipped under a cold chain from the local Metropolis laboratory to the Metropolis Healthcare Ltd. Laboratory in Delhi. A temperature logger was placed with each shipment to record the temperature changes during transportation. Deviation from cold chain temperature was observed during hot periods, especially in regions with tropical or high-temperature weather conditions. In such regions, the field investigators and phlebotomists carried additional ice packs to maintain sample integrity during venous blood specimen collection. The ice packs were also replaced at quality check points: before shipping the samples from the point of collection to the local laboratory, and then from the local laboratory to the central laboratory. At the central laboratory, the serum and plasma samples were aliquoted into 0.5 ml aliquots for long-term storage and performing various biochemical assays. The whole blood assays included Glycosylated hemoglobin (HbA1c), and complete blood cell count (CBC) with differential leucocyte counts. The serum-based assays comprised lipid profile, liver and kidney function tests, thyroid function tests, and other tests, including vitamin B12, folic acid, vitamin D, homocysteine, pro-B-type natriuretic peptide, high-sensitivity C-reactive protein (hsCRP), and lipoprotein-a estimation. We utilized standardized diagnostic criteria of different analytes to classify anemia and its subtypes, such as hemoglobin, serum ferritin, transferrin saturation, vitamin B12, folate, C-reactive protein, and chronic kidney disease estimated using the Chronic Kidney Disease Collaboration (CKD-EPI) equation that utilizes both serum creatinine and cystatin C [45]. The detailed methodology for venous blood sample collection, processing, transportation, with maintenance of the cold chain, and sample storage in LASI-DAD has been published elsewhere [44]. 

### Equipment used for measuring analytes

For measuring analytes relevant to anemia, we employed standardized methods, quality assurance, and equipment for each venous blood sample [44]. Complete blood counts were measured using the Beckman Coulter DxH 8000 instrument. We used the Roche Cobas 8000 (i502 & e801) to quantify serum creatinine, cystatin C, uric acid, vitamin B12, and folic acid. hs-CRP was quantified by nephelometry using Atellica NEPH 630.

### Measures

#### Primary outcome

Anemia was defined as hemoglobin < 13 mg/dl in males and < 12 mg/dl in females [46]. The criteria used to classify anemia into different categories are summarized in Table 1.

**Table 1:** Criteria used to classify anemia in the LASI-DAD study

Classification of anemia	Definitions and measures
**Nutritional anemia**	
Iron deficiency anemia	Serum ferritin level < 30 ng/mL [47] or serum ferritin level of 30–100 ng/mL [48] and transferrin saturation [48] of < 20%.
Vitamin B12 deficiency anemia	Vitamin B12 levels < 200 pg/mL.[49]
Folate deficiency anemia	Serum folate level < 3 ng/dL.[50]
Multiple nutritional deficiencies	More than one nutritional deficiency
**Non-nutritional anemia**	Cases that did not fall under any category of nutritional deficiency anemia
Anemia of chronic disease/inflammation	Serum ferritin levels > 100 ng/ml or hsCRP > 3 mg/dl
Anemia of chronic kidney disease	e-Glomerular Filtration Rate (eGFR) < 60 ml/min
Multiple non-nutritional causes	More than one cause of non-nutritional anemia
Unexplained non-nutritional anemia	Non-nutritional anemia cases that did not fit into the above criteria

#### Covariates

We used age, gender, educational level, urbanicity, and geographical region as covariates. For these analyses, the educational level was recategorized as – no education, less than high school education, completed high school, and college or a higher degree. The individual states were grouped into four regions based on geographical proximity and prevalence of anemia in the individual states to facilitate analysis of regional differences in anemia.

### Statistical analysis

Descriptive statistics were calculated after accounting for sample weights to characterize the study cohort. Two-sample t-tests were used to compare the distribution of continuous variables, such as age, while the χ^2^ test was used to compare the distributions of categorical variables. Weighted frequencies of anemia and its subgroups were calculated after adjusting for sampling weights that account for the complex stratified sampling design. Unconditional multivariate logistic regression models were used (adjusting for sampling weights as a fixed effect) to evaluate the effect of age, sex, regions within India, urban/rural residence, and educational status on anemia. All analyses were conducted using SAS version 9.4.

## Results

The mean age of the participants in this study was 70.2 years, and 50.5% were women. Out of 3252 respondents who consented to a venous blood draw, blood samples from 3009 respondents were deemed sufficient in quantity and quality to conduct bioassay analysis. Among 3009 participants with hemoglobin levels in Wave 2 of LASI-DAD, the overall weighted anemia prevalence was 49.9% (95% CI: 48% − 51.9%) (*n* = 1502). The weighted prevalence of anemia was significantly higher among women as compared to men (53.9% vs. 45.8%; *p* < 0.001). Though the prevalence of anemia was higher in rural areas as compared to urban areas, this difference was not statistically significant, and anemia status did not differ significantly with respondents’ educational status (Table 2).

**Table 2:** Characteristics of the LASI-DAD study population

Sample characteristics	Anemia (*n* = 1502)	No anemia (*n* = 1507)	*P* value
**Age (years)**	70.17 (± 0.18)	69.08 (± 0.15)	< 0.001
**Gender**			< 0.001
Male	682 (45.8%)	806 (54.2%)	
Female	820 (53.9%)	701 (46.1%)	
**Education status**			0.48
No school	752 (50.8%)	728 (49.2%)	
Less than high school	622 (48.3%)	667 (51.7%)	
High school	40 (51.1%)	39 (48.9%)	
College and higher	87 (54.2%)	74 (45.8%)	
Regions*			< 0.001
Region 1	351 (37.8%)	577 (62.2%)	
Region 2	244 (46.8%)	277 (53.2%)	
Region 3	308 (41.0%)	443 (59.0%)	
Region 4	599 (74.1%)	209 (25.9%)	
**Urban/rural setting**			0.09
Urban	420 (47.3%)	467 (52.7%)	
Rural	1082 (51.0%)	1040 (49.0%)	

*Regions:

Region 1: Jammu and Kashmir, Punjab, Uttarakhand, Haryana, Delhi, Rajasthan, Uttar Pradesh, Bihar

Region 2: Maharashtra, Gujarat, and Madhya Pradesh

Region 3: Andhra Pradesh, Karnataka, Tamil Nadu, Pondicherry, Telangana, Kerala

Region 4: Assam, West Bengal, Jharkhand, Odisha, Chhattisgarh

Among LASI-DAD participants with anemia, nutritional causes, which include iron, Vitamin B12, or folate deficiency anemia, characterized 63.6% of the cases (Table 3). Among those with nutritional anemia, isolated iron deficiency anemia was the most common type (51.8%) (Table 3). The second most common cause of nutritional anemia was multiple nutritional deficiencies (26.2%), followed by isolated Vitamin B12 deficiency (12.6%) and isolated folic acid deficiency (9.4%) (Table 3). Among the remaining participants with non-nutritional anemia, chronic disease/inflammation was the most common cause (39.5%) (Table 3). Other causes of non-nutritional anemia were CKD-associated anemia (9.7%) and multiple non-nutritional causes (24.1%) that included both CKD and anemia of chronic disease/inflammation (Table 2). The remaining 146 participants (26.7%) with non-nutritional anemia could not be classified into any identifiable causes of anemia (Table 3).

**Table 3:** Distribution of the different anemia types among the anemic LASI-DAD study population

Anemia type	Anemia (*n* = 1502) (95% CI)	Percentages (95% CI)
**Nutritional Anemia**	**955 (845, 1066)**	**63.6% (61.0%, 66.2%)** ^↨^
Isolated iron deficiency anemia	495 (456, 533)	51.8% (48.4%, 55.1%)
Isolated Vit B12 deficiency anemia	120 (98, 142)	12.6% (10.3%, 14.8%)
Isolated folate deficiency anemia	90 (71, 110)	9.4% (7.5%, 11.4%)
Multiple nutritional deficiency anemia	250 (220, 281)	26.2% (23.3%, 29.2%)
**Non-nutritional anemia**	**547 (457**,** 636)**	**36.4% (33.8%**,**39.0%)** ^↨^
Anemia of chronic disease/inflammation	216 (187, 246)	39.5% (35.2%, 44.1%)
Anemia of chronic kidney disease	53 (39, 67)	9.7% (7.1%, 12.2%)
Multiple non-nutritional causes anemia	132 (111, 151)	24.1% (20.2%, 27.6%)
Unexplained non-nutritional anemia	146 (121, 172)	26.7% (22.7%, 30.9%)

Nutritional anemia was more common in women than men (68.0% vs. 58.2%; *p* = 0.001) (Table 4). Among those with nutritional anemia, isolated iron deficiency anemia was also substantially higher among women as compared to men (60.2% vs. 40.1%; *p* < 0.001) (Table 4). In contrast, isolated vitamin B12 deficiency and isolated folate deficiency were lower in women as compared to men (vitamin B12 deficiency: 9.7% vs. 16.6%; *p* = 0.04 and folate deficiency 5.9% vs. 14.4%; *p* = 0.0008) (Table 4). However, the proportion of men and women with anemia due to multiple nutritional deficiencies was similar (Table 4). Non-nutritional anemia was less common among women than men (32.0% vs. 41.8%; *p* = 0.001). The percentages of CKD-associated anemia and anemia of chronic disease/inflammation were similar among men and women, while anemia due to multiple non-nutritional causes was more common among women (Table 4). The proportion of men with unexplained causes of non-nutritional anemia was substantially higher than that in women (30.2% vs. 22.9%; *p* = 0.0009) (Table 4).

**Table 4:** Sex-stratified distribution of different causes of anemia among those with anemia in LASI-DAD

Anemia type	Men (*n* = 682)	Women (*n* = 820)	*P*- value
**Nutritional anemia**	**397 (58.2%)**	**558 (68.0%)**	**0.001**
Isolated iron deficiency anemia	159 (40.1%)	336 (60.2%)	< 0.0001
Isolated Vit B12 deficiency anemia	66 (16.6%)	54 (9.7%)	0.04
Isolated folate deficiency anemia	57 (14.4%)	33 (5.9%)	0.0008
Multiple nutritional deficiency anemia	115 (29.0%)	135 (24.2%)	0.84
**Non-nutritional anemia**	**285 (41.8%)**	**262 (32.0%)**	**0.001**
Anemia of chronic disease/ inflammation	117 (41.1%)	101 (38.5%)	0.01
Anemia of chronic kidney disease	27 (9.5%)	26 (9.9%)	0.86
Multiple non-nutritional causes anemia	55 (19.3%)	75 (28.6%)	0.02
Unexplained non-nutritional anemia	86 (30.2%)	60 (22.9%)	0.0009

Note: These are column percentages with 95% CI of the column percentages and a significance level of 0.05

The prevalence of anemia was significantly different across the 22 states where participants were enrolled (*p* < 0.0001), with the overall anemia prevalence being > 70% in Assam, West Bengal, Jharkhand, and Odisha, and the overall anemia prevalence being < 30% in Jammu & Kashmir and Haryana. In general, states in East/Northeast India (Region 4) had a substantially higher anemia prevalence (74.1%) than the northern states (Region 1), where the lowest anemia prevalence was observed in India (37.8%) (Table 2). A more detailed evaluation of the subtypes of anemia between the four regions showed that nutritional anemia was significantly lower in Region 4 than in other regions in India (51.6% vs. 69.8%-73.8%; *p* < 0.0001) (Table 5). Among those with nutritional anemia, isolated iron deficiency anemia was slightly lower among participants in Region 4, though this difference was not statistically significant. In contrast, the percentage of isolated folate deficiency among those with nutritional anemia was almost 2–3 times higher in Region 4 as compared to other regions (15.5% vs. 4.4%-7.2%; *p* = 0.0001) (Table 5). Region 4 also had a substantially higher proportion of non-nutritional anemia as compared to other regions (48.4% vs. 26.2%-30.2%; *p* < 0.0001) (Table 5). Though there were no differences in the distribution of non-nutritional anemia types among the regions, the number of unexplained cases of non-nutritional anemia was slightly higher in Region 4 as compared to other regions (32.1% vs. 18.8%-22.6%; *p* = 0.05) (Table 5).

**Table 5:** Region-stratified distribution of different causes of anemia among those with anemia in LASI-DAD

Anemia type	Region 1 (*n* = 351)	Region 2 (*n* = 244)	Region 3 (*n* = 308)	Region 4 (*n* = 599)	*P*- value
**Nutritional Anemia**	250 (71.2%)	180 (73.8%)	215 (69.8%)	309 (51.6%)	< 0.0001
Isolated iron deficiency anemia	70 (28.0%)	43 (23.9%)	66 (30.7%)	70 (22.7%)	0.35
Isolated Vit B12 deficiency anemia	118 (47.2%)	99 (55.0%)	118 (54.9%)	160 (51.8%)	0.003
Isolated folate deficiency anemia	18 (7.2%)	8 (4.4%)	15 (7.0%)	48 (15.5%)	0.0001
Multiple nutritional deficiency anemia	44 (17.6%)	30 (16.7%)	16 (7.4%)	31 (10.0%)	0.19
**Non-nutritional anemia**	101 (28.8%)	64 (26.2%)	93 (30.2%)	290 (48.4%)	< 0.0001
Anemia of chronic disease/inflammation	41 (40.6%)	27 (42.2%)	36 (38.7%)	112 (38.6%)	0.95
Anemia of chronic kidney disease	11 (10.9%)	6 (9.4%)	8 (8.6%)	29 (10.0%)	0.90
Multiple non-nutritional causes anemia	28 (27.7%)	19 (29.7%)	28 (30.1%)	56 (19.3%)	0.08
Unexplained non-nutritional causes	21 (20.8%)	12 (18.8%)	21 (22.6%)	93 (32.1%)	0.05

Notes: Percentages are column percentages, at a significance level of 0.05

* Regions:

Region 1: Jammu and Kashmir, Punjab, Uttarakhand, Haryana, Delhi, Rajasthan, Uttar Pradesh, Bihar

Region 2: Maharashtra, Gujrat, and Madhya Pradesh

Region 3: Andhra Pradesh, Karnataka, Kerala, Tamil Nadu, Pondicherry, Telangana

Region 4: Assam, West Bengal, Jharkhand, Odisha, Chhattisgarh

Multivariate analyses showed that participant age, sex, and region of residence continue to be independently associated with anemia status.

Between waves 1 and 2, 236 participants developed incident anemia in wave 2. The distribution of the incident and prevalent anemia cases was very similar in wave 2, with incident anemia being similar among women and men (51.3% vs. 48.7%; *p* = 0.20). There were no substantial rural/urban differences in anemia incidence. Regions 2 and 3 contributed to 18.2%-20.8% of cases of incident anemia, while Region 4 contributed to 28.4% of cases. Region 1 contributed a third of all incident anemia cases (33.3%) (*p* < 0.0001). Among those with incident anemia, nutritional anemia was more common in women (63.5%) than men (47.8%) (*p* = 0.02) (See Supplementary Table 1, Additional File 1). There were no regional differences in the distribution of incident anemia in wave 2 (See Supplementary Table 2, Additional File 2).

## Discussion

The LASI-DAD study confirms the high national prevalence of anemia among older adults aged 60 years and above in India. We observed that nearly half of the older adults had anemia, with the overall and nutritional anemia prevalences being higher among women. This is the first study to evaluate the causes of anemia nationally and at the regional level. We show that there are substantial regional differences in anemia prevalence, with a higher prevalence in Eastern/Northeastern states, which can be attributed to a higher prevalence of non-nutritional anemia in these states.

The finding that nearly half of the older adult population in India is anemic supports previous observations that have shown a high prevalence of anemia (68.3%) among older adults in India [2]. Previous studies on older adults in India have shown highly variable estimates for anemia prevalence, ranging from 20.6% to 96% [21, 22, 51–52]. However, most studies on the geriatric population in India report prevalence estimates of more than 50% (ranging from 57% to 96%) [15–21, 24–28, 24–35, 53–55] Some studies have reported anemia prevalence similar to ours ranging from 45.5% to 49.6% [23, 30, 31]. Heterogeneity in prevalence estimates can be attributed to the variety of laboratory methods used to estimate anemia, smaller sample size (*n* = 60 to *n* = 982), hospital/institution-based samples, limited participant inclusivity (studies with only male or female participants), a convenience sampling design and/or sampling limited to individual states within India that limit the comparability and extrapolation of their results to a nationally representative study. Our study addresses all these limitations as we employed a two-stage stratified sampling technique nested with the Longitudinal Aging Study of India (LASI) cohort which allowed us to provide nationally representative prevalence estimates of anemia among people aged 60 years and above in India. Furthermore, we used standardized automated methods in a central laboratory for hemoglobin estimation and measuring other relevant analytes to determine the causes of anemia for all venous blood samples collected across India. A particular strength of this study is the ability to classify the underlying causes of anemia in a nationally representative population. Many community-based studies have reported the prevalence of anemia; however, only a few have studied the underlying causes [20–22]. In contrast, most studies that determined the underlying causes of anemia were hospital-based [55–63]. Our findings are consistent with previous reports that indicate that nutritional deficiency is the most common cause of anemia among older adults [22, 60, 62]. Similar to the variability seen in overall anemia prevalence in previous studies, there is considerable heterogeneity in the estimates of nutritional anemia in published literature. Studies have reported that nutritional anemia contributed to 26.8% to 56.8% of anemia cases [21, 22, 48, 49, 59–66]. Differences in methodology that allowed the identification of limited nutritional deficiencies only, variations in diagnostic criteria, a smaller sample size (*n* = 70 to *n* = 100), and recruitment of participants with pre-existing medical conditions are possible explanations for the observed differences from previous studies [56, 57, 59, 60, 62, 63]. 

Among the nutritional anemias, the present study found that iron deficiency anemia was the most common type, followed by vitamin B12 and folate deficiency anemia. In addition, anemia due to multiple nutritional deficiencies was the second most common form of nutritional anemia. These results are all largely consistent with previous studies that have evaluated different causes of nutritional anemia [55–62]. However, as stated previously, there is considerable heterogeneity in these estimates from previous studies due to small sample sizes [56–60, 62], hospital-based sampling [55–62], differences in criteria used to define specific nutritional deficiencies, such as using serum ferritin levels < 15 ng/mL to define isolated iron deficiency anemia [22], which is lower than the cut-off (< 30 ng/mL) used in our analysis and the lack of comprehensive evaluation of all nutritional deficiencies assessed in the studies [20, 21, 55, 59, 63]. An additional source of heterogeneity when identifying different types of nutritional anemia was using red blood cell (RBC) indices such as mean corpuscular volume (MCV) and RBC size to classify nutritional anemia [56, 63]. Given the high proportion of multiple nutritional deficiencies in the LASI-DAD population with anemia, the use of RBC indices such as RBC size and MCV to classify anemias resulted in an inability to classify a majority of individuals with anemia (data not shown), and hence RBC indices were not used in the categorization of anemia in this study.

The proportion of cases attributed to non-nutritional anemia in this study was also similar to previously published estimates [58, 60–62]. While other studies have published higher estimates of non-nutritional anemia, these studies were hospital-based studies where one would expect a higher prevalence of anemia due to pre-existing chronic diseases/inflammation among the recruited participants [55–57, 59, 63]. Anemia of chronic disease/inflammation was the most common cause of non-nutritional anemia and contributed to 40% of the cases. Previous community-based studies that have reported anemia prevalence have not comprehensively evaluated non-nutritional causes of anemia. However, the estimates of anemia of chronic disease/inflammation reported by hospital-based studies vary from our findings to some extent (21% to 56%)[55–63]. Our study found that 3.5% of all anemia cases were related to CKD. In other studies, CKD accounted for 11–20% of all anemia cases [56, 58, 60, 61]. The higher proportion of anemia related to chronic disease/inflammation and CKD in previous studies may be attributed to the likelihood of pre-existing chronic disease/inflammatory and risk factors such as diabetes, hypertension, and cardiovascular diseases among the participants recruited in hospital-based studies as compared to those in the community. Our study was able to categorize 90.3% of all anemia cases. Our findings are consistent with a few previous studies that reported a prevalence of unexplained anemia ranging from 8–11% [55, 57–61]. Though two previous studies reported a higher percentage (20-21.6%) of unexplained anemia [56, 62]), the comprehensive biochemical and hematological assessments used in LASI-DAD allowed us to identify a vast majority of the causes of anemia in this study.

Though the prevalence of anemia in our study was higher in rural areas than in urban areas, the difference was not statistically significant. These results are consistent with previous studies that have reported a similar trend [33, 35]. The most surprising finding in this study was the high prevalence of anemia in Region 4 (Assam, West Bengal, Jharkhand, Odisha, and Chhattisgarh) as compared to other regions in India. This finding is consistent with another study using data of 61,481 men from rural areas aged 15–54 years from the National Family Health Survey (NFHS)-5 [40], and showed that the districts with high anemia prevalence were from the eastern region including West Bengal, Odisha, Chhattisgarh, and Assam. However, NFHS also showed that the union territories of Jammu & Kashmir and Ladakh also had a high prevalence of anemia [40]. The study also reported the lowest prevalences from the northern states (Uttarakhand and Himachal Pradesh), some districts of north-eastern states (Nagaland and Mizoram), and southern states (Tamil Nadu and Karnataka). In contrast to the findings from our study, another study, which utilized the LASI data and asked people to self-report diagnosis of anemia within the last 2 years found [66] anemia prevalence to be highest in Region 1 (33.4%) followed by Region 4 (23.24%). However, since the prevalence estimate in the LASI study was based on self-reported cases of anemia, which are very sensitive to recall bias, knowledge and awareness regarding anemia, healthcare utilization, and completion of anemia treatment. In addition, the higher prevalence of self-reported anemia in LASI in Region 1 is likely biased as regions with relatively better healthcare facilities have easier accessibility to screening tests than others. Additional studies [27, 54, 59] from the eastern region of India have reported anemia prevalence ranging from 65% to 89.5%, which is consistent with the estimates from our study. Additional analysis of the distribution of different types of anemia in the four regions showed that non-nutritional anemia was significantly higher in Region 4 as compared to other regions in India. The reason for this finding is unclear at this time. Analysis of the prevalence of self-reported stroke, heart disease, and chronic kidney disease did not show any meaningful differences between the regions. Notably, the proportion of individuals with unexplained anemia was significantly higher in Region 4 (32.1%; *p* = 0.05) as compared to other regions (18.8%-22.6%). Given the higher prevalence of hemoglobinopathies in Eastern/North Eastern parts of India as compared to other regions [67], future studies that evaluate the prevalence of hemoglobinopathies in different states within India may provide additional insights into the possible reasons for the higher prevalence of anemia in Eastern/Northeastern states in India. The observed geographical differences can potentially have major implications for anemia control in India. Though the focus on reducing iron deficiency anemia is a nationwide goal and is supported by data from LASI-DAD and other studies, there may be an additional need to address the different causes of anemia using specific strategies in individual states to reduce anemia prevalence.

In wave 1 of LASI-DAD, the prevalence of anemia among older adults (*n* = 2758) was found to be about 40% [68]. The prevalence increased to 49.9% over the years among the LASI-DAD study participants. Incident anemia cases followed a pattern that was very similar to that observed with prevalent anemia with nutritional anemia (and iron deficiency anemia) being the most frequent cause of anemia.

Major strengths of this study include the nationally representative sampling design, high quality of the samples assayed, standardization of protocols, and representation of the geriatric population from both rural and urban communities on a national level. However, our study has certain limitations. Though we were able to identify iron deficiency anemia as the major cause of anemia in this population, this study could not identify the potential sources of iron deficiency. Insufficient dietary intake, occult blood loss due to malignancy or parasitic infections, and malabsorption are frequently observed among older adults. A major limitation of the LASI-DAD study was that we did not collect additional information to identify the causes of IDA in the study population. About 10% of the anemia cases were due to unexplained causes. One of the major causes that was not evaluated in this study was the contribution of hemoglobinopathies to anemia in this population. A recent study in North India showed that the prevalence of hemoglobinopathies was 8.5% and was almost three times higher in men (18.9%) as compared to women (6.9%). Therefore, evaluating hemoglobinopathies in this population may help explain the unexplained causes of anemia.

## Conclusion

Our study findings have shown that approximately 50% of older adults are suffering from anemia, with nutritional deficiencies accounting for two-thirds of all anemia cases. This is a rapidly growing public health concern, especially for those with iron deficiency, anemia of chronic disease, and multiple underlying causes. The risk of anemia increases with age and the two waves of LASI-DAD show that the overall prevalence of anemia increased by 10%. Health policies to curb the anemia epidemic in India should also include older adults as a highly vulnerable group, besides children, adolescents, and women in the reproductive age group. India is experiencing a rapid epidemiological transition in disease patterns and will see an exponential increase in the population size of older adults in the coming decades [69]. The study results highlight the need to expand national anemia control programs, such as the Anemia Mukt Bharat, to include anemia control in older adults as well and/or integrate it into the National Program for the Health Care of the Elderly (NPHCE).

In addition to national anemia control programs focused on older adults, given the regional differences in prevalence and causes of anemia, the results of this study suggest that state-level anemia screening and treatment programs may be most effective in reducing the burden of anemia among older adults. Our study highlights the high overall prevalence of cases in the states of Eastern and North-Eastern regions, with a significantly higher proportion of anemia cases in these regions being attributed to non-nutritional causes. Specifically, these areas showed a higher prevalence of unexplained anemia, suggesting that factors such as hemoglobinopathies may account for a greater proportion of anemia in these states. If confirmed in other studies, this finding would suggest that targeted screening for hemoglobinopathies may be more effective in reducing anemia prevalence in Region 4 rather than a focus on nutritional anemia alone. The lower overall prevalence of anemia in Region 1 (North India) is an encouraging sign. Though the relative proportion of nutritional and non-nutritional causes of anemia was similar in Region 1 compared to Regions 2 and 3, the lower overall anemia prevalence in Region 1 suggests that national efforts to reduce anemia may be more effective in Region 1 than in other regions. A detailed understanding of the factors resulting in lower anemia prevalence in Region 1 may help design and implement effective anemia control programs in the rest of the county. This study highlights the importance of identifying and treating the specific causes of anemia that differ across states in India. This study supports that continued focus on nutritional anemia in general and particularly iron deficiency anemia in anemia control programs, as these are the dominant causes of anemia in almost all regions in India. However, adding additional region-specific components to the anemia prevention program may improve the program’s effectiveness.

In our study, 10% of the anemia cases were unexplained due to data limitations. It indicates the need for conducting pilot screenings for hemoglobinopathies, especially in high-prevalence states and regions, to provide a comprehensive map of other causes of anemia. The lack of serum ferritin measurements in LASI-DAD wave 1 did not allow us to estimate the change in percentage of nutritional and non-nutritional anemia across the two waves. Through our study, we demonstrate the feasibility of gathering high-quality, blood-based biomarker data through a logistically strong home-based collection and meticulous quality assurance. Overall, our study highlights the need to design health programs that are inclusive of the healthcare needs of older adults and are well-equipped to cater to the underserved aging population of India.

## Supplementary Information

Below is the link to the electronic supplementary material.

Supplementary Material 1

## Data Availability

The dataset(s) supporting the conclusions of this article can be accessed from the [Gateway to Global Aging Data Website](https://g2aging.org/?section=lasi-dad-download) [70].

## Abbreviations

LASI-DAD: Harmonized Diagnostic Assessment of Dementia for the Longitudinal Aging Study in India
AIIMS: All India Institute of Medical Sciences
HbA1c: Glycosylated hemoglobin
CBC: Complete Blood Count
hsCRP: High-sensitivity C-reactive protein
CKD-EPI: Chronic Kidney Disease Epidemiology Collaboration
eGFR: e-Glomerular Filtration Rate
RBC: Red Blood Cell
MCV: Mean Corpuscular Volume
CKD: Chronic Kidney Disease
NFHS: National Family Health Survey

## References

[1] Gardner WM, Razo C, McHugh TA, et al. Prevalence, years lived with disability, and trends in anemia burden by severity and cause, 1990–2021: findings from the global burden of disease study 2021. Lancet Haematol. 2023;10(9):e713–34. 10.1016/S2352-3026(23)00160-6. 37536353 10.1016/S2352-3026(23)00160-6 PMC10465717

[2] Daniel RA, Ahamed F, Mandal S, Lognathan V, Ghosh T, Ramaswamy G. Prevalence of anemia among the elderly in india: evidence from a systematic review and meta-analysis of cross-sectional studies. Cureus 15(7):e42333. 10.7759/cureus.42333 10.7759/cureus.42333 PMC10443921 37614252

[3] Chaparro CM, Suchdev PS. Anemia epidemiology, pathophysiology, and etiology in low- and middle-income countries. Ann N Y Acad Sci. 2019;1450(1):15–31. 10.1111/nyas.14092. 31008520 10.1111/nyas.14092 PMC6697587

[4] Penninx BWJH, Pahor M, Cesari M, et al. Anemia is associated with disability and decreased physical performance and muscle strength in the elderly. J Am Geriatr Soc. 2004;52(5):719–24. 10.1111/j.1532-5415.2004.52208.x. 15086651 10.1111/j.1532-5415.2004.52208.x

[5] Hong CH, Falvey C, Harris TB, et al. Anemia and risk of dementia in older adults. Neurology. 2013;81(6):528–33. 10.1212/WNL.0b013e31829e701d. 23902706 10.1212/WNL.0b013e31829e701d PMC3775683

[6] Wolters FJ, Zonneveld HI, Licher S, et al. Hemoglobin and anemia in relation to dementia risk and accompanying changes on brain MRI. Neurology. 2019;93(9):e917–26. 10.1212/WNL.0000000000008003. 31366722 10.1212/WNL.0000000000008003 PMC6745727

[7] Duh MS, Mody SH, Lefebvre P, Woodman RC, Buteau S, Piech CT. Anaemia and the risk of injurious falls in a community-dwelling elderly population. Drugs Aging. 2008;25(4):325–34. 10.2165/00002512-200825040-00005. 18361542 10.2165/00002512-200825040-00005

[8] Palmer K, Vetrano DL, Marengoni A, et al. The relationship between anaemia and frailty: A systematic review and Meta-Analysis of observational studies. J Nutr Health Aging. 2018;22(8):965–74. 10.1007/s12603-018-1049-x.30272101 10.1007/s12603-018-1049-x

[9] Culleton BF, Manns BJ, Zhang J, Tonelli M, Klarenbach S, Hemmelgarn BR. Impact of anemia on hospitalization and mortality in older adults. Blood. 2006;107(10):3841–6. 10.1182/blood-2005-10-4308. 16403909 10.1182/blood-2005-10-4308

[10] Karoopongse E, Srinonprasert V, Chalermsri C, Aekplakorn W. Prevalence of anemia and association with mortality in community-dwelling elderly in Thailand. Sci Rep. 2022;12:7084. 10.1038/s41598-022-10990-7. 35490162 10.1038/s41598-022-10990-7 PMC9056501

[11] Little M, Zivot C, Humphries S, Dodd W, Patel K, Dewey C. Burden and determinants of anemia in a rural population in South india: A Cross-Sectional study. Anemia. 2018;2018:7123976. 10.1155/2018/7123976. 30112198 10.1155/2018/7123976 PMC6077670

[12] Stauder R, Thein SL. Anemia in the elderly: clinical implications and new therapeutic concepts. Haematologica. 2014;99(7):1127–30. 10.3324/haematol.2014.109967. 24986873 10.3324/haematol.2014.109967 PMC4077071

[13] Alvarez-Payares JC, Rivera-Arismendy S, Ruiz-Bravo P et al. Unexplained anemia in the elderly. Cureus 13(11):e19971. 10.7759/cureus.19971 10.7759/cureus.19971 PMC8714032 34984131

[14] Kaur D, Rasane P, Singh J, et al. Nutritional interventions for elderly and considerations for the development of geriatric foods. Curr Aging Sci. 2019;12(1):15–27. 10.2174/1874609812666190521110548. 31109282 10.2174/1874609812666190521110548 PMC6971894

[15] Pathania A, Haldar P, Kant S, Gupta SK, Pandav CS, Bachani D. Prevalence of anemia among elderly persons residing in old age homes in National capital Territory, Delhi, India. Indian J Public Health. 2019;63(4):288. 10.4103/ijph.IJPH_412_18. 32189646 10.4103/ijph.IJPH_412_18

[16] Renjini BA, Raj A, Krishnendu VK, Rajiv M, Divyamol S, Rakesh PS. High prevalence of malnutrition and anemia among elderly at old age homes in Kerala, India. | J Med Allied Sci | EBSCOhost. 10.5455/jmas.7814

[17] Shrivastava SR, Hippargi SB, Ambali AP, Yelikar BR. Patterns anemia geriatric age group. 2013;2(1).

[18] Soni PN, Jawale RB, Soni SP. Study of anemia in geriatric population: a hospital based study in Marathwada region, Maharashtra, India. Int J Adv Med. 2016;3(2):197–9. 10.18203/2349-3933.ijam20161069.

[19] Singh T, Nagesh S, Ray TK. Magnitude and correlates of anemia in elderly women of a resettlement colony of Delhi. J Midlife Health. 2018;9(1):21–5. 10.4103/jmh.JMH_57_17. 29628724 10.4103/jmh.JMH_57_17 PMC5879843

[20] FullUniWP. Aging medicine and healthcare | hemoglobin, folate and vitamin B12 status of economically deprived elderly women. December 31, 2017. Accessed July 18, 2024. https://www.agingmedhealthc.com/?p=21074

[21] Gonmei Z, Dwivedi S, Toteja GS, Singh K, Vikram NK, Bansal PG. Anemia and vitamin B12 deficiency in elderly. Asian journal of pharmaceutical and clinical research. Published Online January. 2018;1:402–4. 10.22159/ajpcr.2018.v11i1.23750.

[22] Vadakattu SS, Ponday LR, Nimmathota A, et al. Prevalence of nutritional anemia and hyperhomocysteinemia in urban elderly. Indian J Clin Biochem. 2019;34(3):330–5. 10.1007/s12291-018-0756-8. 31391724 10.1007/s12291-018-0756-8 PMC6660523

[23] Lamba R, Agarwal A, Rana R, Agarwal V. Prevalence of anemia and its correlates among elderly population of an urban slum in meerut. J Indian Acad Geriatr. 2019;15(3):109. 10.35262/jiag.v15i3.109-114.

[24] Retnakumar C, Chacko M, Ramakrishnan D, George LS, Krishnapillai V. Prevalence of anemia and its association with dietary pattern among elderly population of urban slums in Kochi. J Family Med Prim Care. 2020;9(3):1533–7. 10.4103/jfmpc.jfmpc_1113_19. 32509645 10.4103/jfmpc.jfmpc_1113_19 PMC7266240

[25] Agrawal S, Deo J, Verma AK, Kotwal A. Geriatric health: need to make it an essential element of primary health care. Indian J Public Health. 2011;55(1):25. 10.4103/0019-557X.82540. 21727677 10.4103/0019-557X.82540

[26] P SB, K CT. A study to assess the prevalence of anemia and activities of daily living among elderly population residing in a South Indian rural community. Int J Community Med Public Health. 2016;3(2):437–41. 10.18203/2394-6040.ijcmph20160427

[27] Maiti S, Ghosh A, Ali K, Ghosh D, Paul S. Prevalence of anaemia among the male population aged 60 years and above in rural area of Paschim Medinipur, West Bengal, India. Health Renaissance. 2013;11(1):23–6. 10.3126/hren.v11i1.7597.

[28] Punia A, Punia M, Sheoran B, et al. To study the prevalence of microscopic blood pictures and anemia among elderly in rural areas of a District in Haryana, India. Internet J Epidemiology. 2015;13:1. 10.5580/IJE.25405

[29] Paul SS, Abraham VJ. How healthy is our geriatric population? A community-based cross-sectional study. J Family Med Prim Care. 2015;4(2):221–5. 10.4103/2249-4863.154653. 25949971 10.4103/2249-4863.154653 PMC4408705

[30] Agarwalla R, Saikia AM, Parashar M, Pathak R, Islam F. Assessment of prevalence of anemia in and its correlates among community-dwelling elderly of Assam, india: A cross-sectional study. Int J Nutr Pharmacol Neurol Dis. 2016;6(1):23. 10.4103/2231-0738.173788.

[31] Kant S, Kumar R, Malhotra S, Kaur R, Haldar P. Prevalence and determinants of anemia among adult males in a rural area of Haryana, India. J Epidemiol Glob Health. 2019;9(2):128–34. 10.2991/jegh.k.190513.001. 31241871 10.2991/jegh.k.190513.001 PMC7310746

[32] Gupta A, Ramakrishnan L, Pandey RM, et al. Risk factors of anemia amongst elderly population living at high-altitude region of India. J Family Med Prim Care. 2020;9(2):673–82. 10.4103/jfmpc.jfmpc_468_19. 32318402 10.4103/jfmpc.jfmpc_468_19 PMC7113975

[33] Swami H, Bhatia V, Dutt R, Bhatia S. A community based study of the morbidity profile among the elderly in Chandigarh, India.

[34] Maninder K, Kochar GK. Burden of anaemia in rural and urban Jat women in Haryana state, India. Malays J Nutr. 2009;15(2):175–84. 22691815

[35] Bharati DR, Pal R, Rekha R, Yamuna TV, Kar S, Radjou AN. Ageing in Puducherry, South india: an overview of morbidity profile. J Pharm Bioallied Sci. 2011;3(4):537–42. 10.4103/0975-7406.90111. 22219588 10.4103/0975-7406.90111 PMC3249702

[36] Kapil U, Kapil R, Gupta A. National iron plus initiative: current status & future strategy. Indian J Med Res. 2019;150(3):239–47. 10.4103/ijmr.IJMR_1782_18. 31719294 10.4103/ijmr.IJMR_1782_18 PMC6886130

[37] LASI_India_Executive_Summary.pdf. Accessed July 18. 2024. https://www.iipsindia.ac.in/sites/default/files/LASI_India_Executive_Summary.pdf

[38] Roberts DJ, Matthews G, Snow RW, Zewotir T, Sartorius B. Investigating the Spatial variation and risk factors of childhood anaemia in four sub-Saharan African countries. BMC Public Health. 2020;20(1):126. 10.1186/s12889-020-8189-8. 31996196 10.1186/s12889-020-8189-8 PMC6990548

[39] Sharma H, Singh SK, Srivastava S. Socio-economic inequality and Spatial heterogeneity in anaemia among children in india: evidence from NFHS-4 (2015–16). Clin Epidemiol Global Health. 2020;8(4):1158–71. 10.1016/j.cegh.2020.04.009.

[40] Singh A, Ram S, Chandra R, Tanti A, Singh S, Kundu A. A district-level Geospatial analysis of anaemia prevalence among rural men in India, 2019-21. Int J Equity Health. 2024;23:9. 10.1186/s12939-023-02089-w. 38243230 10.1186/s12939-023-02089-w PMC10799465

[41] Lee J, Khobragade PY, Banerjee J, et al. Design and methodology of the longitudinal aging study in India-Diagnostic assessment of dementia (LASI-DAD). J Am Geriatr Soc. 2020;68(Suppl 3):S5–10. 10.1111/jgs.16737. 32815602 10.1111/jgs.16737 PMC7503220

[42] Khobragade PY, Petrosyan S, Dey S, Dey AB, Lee J, Team the LDA. Design and methodology of the harmonized diagnostic assessment of dementia for the longitudinal aging study in India: Wave 2. Journal of the American Geriatrics Society. (n/a). 10.1111/jgs.19252 10.1111/jgs.19252 PMC11907746 39482079

[43] Lee J, Banerjee J, Khobragade PY, Angrisani M, Dey AB. LASI-DAD study: a protocol for a prospective cohort study of late-life cognition and dementia in India. BMJ Open. 2019;9(7):e030300. 10.1136/bmjopen-2019-030300. 31371300 10.1136/bmjopen-2019-030300 PMC6677961

[44] Dhankhar A, Khobragade P, Banerjee J, et al. Methodology of community-based venous blood specimen collection for the harmonized diagnostic assessment of dementia for the longitudinal aging study in India (LASI-DAD): wave 2. PLoS ONE. 2025;20(7):e0326917. 10.1371/journal.pone.0326917. 40743219 10.1371/journal.pone.0326917 PMC12312927

[45] Inker LA, Schmid CH, Tighiouart H, et al. Estimating glomerular filtration rate from serum creatinine and Cystatin C. N Engl J Med. 2012;367(1):20–9. 10.1056/NEJMoa1114248. 22762315 10.1056/NEJMoa1114248 PMC4398023

[46] World Health Organization, ed. Guideline on haemoglobin cutoffs to define anaemia in individuals and populations. World Health Organization; 2024. 38530913

[47] Al-Naseem A, Sallam A, Choudhury S, Thachil J. Iron deficiency without anaemia: a diagnosis that matters. Clin Med (Lond). 2021;21(2):107–13. 10.7861/clinmed.2020-0582. 33762368 10.7861/clinmed.2020-0582 PMC8002799

[48] Barney J, Moosavi L. Iron. In: StatPearls. StatPearls Publishing. Accessed July 18, 2024. http://www.ncbi.nlm.nih.gov/books/NBK542171/

[49] Ankar A, Kumar A. Vitamin B12 Deficiency. In: StatPearls. StatPearls Publishing. Accessed July 18, 2024. http://www.ncbi.nlm.nih.gov/books/NBK441923/28722952

[50] Singh G, Hamdan H, Singh V. Clinical utility of serum folate measurement in tertiary care patients: argument for revising reference range for serum folate from 3.0 ng/mL to 13.0 ng/mL. Pract Lab Med. 2015;1:35–41. 10.1016/j.plabm.2015.03.005. 28932797 10.1016/j.plabm.2015.03.005 PMC5597708

[51] Ashwini Aithal P, Binti Amber A, Wen Hao C, et al. Incidence and factors associated with anemia among the geriatric population at a tertiary care hospital in Southern India. Rmj. 2023;80(1):29–34. 10.4314/Rmj.v80i1.4.

[52] Malhotra D, Kabra D, Bhayya D, Malhotra R. Prevalence and correlates of anemia among elderly population of rural nalgonda: a cross-sectional analytic study. Public Health Review: Int J Public Health Res. 2016;3:168–73. 10.17511/ijphr.2016.i4.06.

[53] Thotakura M, Bharathi VM, Staynarayana YK. Prevalence of morphological patterns of anemia in geriatric population. Int J Clin Diagn Pathol. 2021;4(3):178–80. 10.33545/pathol.2021.v4.i3c.427.

[54] Debnath A, Rehman T, Ghosh T, Kaur A, Ahamed F. Prevalence of anemia among elderly population residing in an urban area of West bengal: A Community-Based Cross-Sectional analytical study. Indian J Community Med. 2022;47(4):604–8. 10.4103/ijcm.ijcm_522_22. 36742972 10.4103/ijcm.ijcm_522_22 PMC9891053

[55] Kadhiravan E, Devi A, Mohan R, Sindhuri R, Abdula S, Devi DSK. Anemia in elderly Patients ≥ 65 years of age: a Hospital-based Cross-sectional study. J Indian Acad Geriatr. 2023;19(1):19. 10.4103/jiag.jiag_57_22.

[56] Characteristics of anemia in patients above 60 years of age attending a tertiary care hospital.pdf. Accessed August 14. 2024. https://journal-innovations.com/assets/uploads/doc/62129-23263.pdf

[57] Krishnamurthy S, Kumar B, Thangavelu S. Clinical and hematological evaluation of geriatric anemia. J Family Med Prim Care. 2022;11(6):3028–33. 10.4103/jfmpc.jfmpc_2239_21. 36119248 10.4103/jfmpc.jfmpc_2239_21 PMC9480756

[58] Sharma D, Suri V, Pannu AK, et al. Patterns of geriatric anemia: A hospital-based observational study in North India. J Family Med Prim Care. 2019;8(3):976–80. 10.4103/jfmpc.jfmpc_450_18. 31041236 10.4103/jfmpc.jfmpc_450_18 PMC6482803

[59] Bisht N, Sofia SR, Neki NS. Prevalence and pattern of anemia in elderly: a hospital based study. Int J Curr Res Med Sci. 2017;3(6):27–35. 10.22192/ijcrms.2017.03.06.004

[60] Sherawat M, Mittal V, Arora S, Kumar R. Patterns of anaemia in elderly patients in relation with RBC indices. Int J Curr Res Rev. 2021;13:78–82. 10.31782/IJCRR.2021.13306.

[61] Agarwal N, Kumar C, Jose M, et al. Haematological patterns of anaemia in geriatric patients and its etiology: A study of 500 cases. Journal Pharm Res International Published Online March. 2022;16:17–25. 10.9734/JPRI/2022/v34i24B35938.

[62] Shastri M, Kotru M, Raizada A, Mahajan B, Jain R, Sikka M. Inflammatory markers in geriatric anemia: A study from North India. J Family Med Prim Care. 2023;12(8):1663. 10.4103/jfmpc.jfmpc_2443_22. 37767440 10.4103/jfmpc.jfmpc_2443_22 PMC10521830

[63] Raisinghani N, Kumar S, Acharya S, Gadegone A, Pai V. Does aging have an impact on hemoglobin? Study in elderly population at rural teaching hospital. J Family Med Prim Care. 2019;8(10):3345. 10.4103/jfmpc.jfmpc_668_19. 31742166 10.4103/jfmpc.jfmpc_668_19 PMC6857363

[64] Nutritional Anemia in Geriatric Population. Accessed August 13, 2024. https://www.wjoanemia.com/abstractArticleContentBrowse/WJOA/18592/JPJ/fullText

[65] Srivastava T, Negandhi H, Neogi SB, Sharma J, Saxena R. Methods for hemoglobin estimation: a review of what works. Journal of Hematology and Transfusion. Published online November 3, 2014. Accessed July 18, 2024. https://www.jscimedcentral.com/article/Methods-for-Hemoglobin--Estimation%3A-A-Review-of-%E2%80%9CWhat--Works%E2%80%9D

[66] Halder P, Verma M, Pal S, et al. Association of anaemia with indoor air pollution among older Indian adult population: multilevel modelling analysis of nationally representative cross-sectional study. BMC Geriatr. 2024;24:567. 10.1186/s12877-024-05171-2. 38951755 10.1186/s12877-024-05171-2 PMC11218345

[67] Bansal C, Chopra G, Singh KK, Singh R, Jatale R, Ramchandran S. Usefulness of high performance liquid chromatography and red cell indices as a screening tool for diagnosing haemoglobinopathies: A retrospective observational study from North India. Asian Hematol Res Journal Published Online April. 2024;2:27–40.

[68] Winchester LM, Newby D, Ghose U et al. Anemia, hemoglobin concentration and cognitive function in the longitudinal ageing study in India-harmonized diagnostic assessment of dementia (LASI-DAD) and the health and retirement study. *medRxiv*. Published online January 23, 2024:2024.01.22.24301583. 10.1101/2024.01.22.24301583

[69] Lee J, Meijer E, Langa KM, et al. Prevalence of dementia in india: National and state estimates from a nationwide study. Alzheimer’s Dement. 2023;19(7):2898–912. 10.1002/alz.12928. 36637034 10.1002/alz.12928 PMC10338640

[70] Jinkook Lee AB, Dey S, Petrosyan P, Khobragade A, Weerman S, Chien J, Banerjee A, Gross E, Nichols P, Crimmins HE, Angrisani M, Langa K, Varghese M, Sharmistha Dey. Harmonized diagnostic assessment of dementia for the longitudinal aging study in India (LASI-DAD) Wave 2 Version A Data. 2025.https://g2aging.org/survey/get_data. Produced and distributed by the University of Southern California with funding from the National Institute on Aging (R01AG051125, U01AG065958).

