# Supplementary material for: Prevalence and causes of anemia among older adults in India: findings from wave 2 of the Harmonized Diagnostic Assessment of Dementia for the Longitudinal Aging Study in India (LASI-DAD)
Source: Int J Equity Health. 2025 Nov 14;24:315. doi: 10.1186/s12939-025-02671-4 (PMC12619410; doi:10.1186/s12939-025-02671-4)
Supplement: Supplementary file 1 — Supplementary Material 1 [file 12939_2025_2671_MOESM1_ESM.docx]

**Supplementary Table 1:** Sex-stratified distribution of different causes of incident anemia among those with anemia in LASI-DAD

| **Anemia type** | **Male [n (%) (n=115)]** | **Female [n (%)) (n=121)]** | **p-value** |
| --- | --- | --- | --- |
| **Nutritional Anemia** | 55 (47.8%) | 77 (63.6%) | 0.02 |
| Isolated iron deficiency anemia | 26 (47.3%) | 39 (50.6%) | 0.72 |
| Isolated Vit B12 deficiency anemia | 8 (14.5%) | 10 (13.0%) | 0.97 |
| Isolated folate deficiency anemia | 11 (20.0%) | 5 (6.5%) | 0.03 |
| Multiple nutritional deficiency anemia | 10 (18.2%) | 23 (29.9%) | 0.20 |
| **Non-nutritional anemia** | 60 (52.2%) | 44 (36.4%) | 0.02 |
| Anemia of chronic disease/inflammation | 25 (41.7%) | 16 (36.4%) | 0.53 |
| Anemia of chronic kidney disease | 8 (13.3%) | 3 (6.8%) | 0.17 |
| Multiple non-nutritional causes anemia | 9 (15.0%) | 13 (29.5%) | 0.10 |
| Unexplained non-nutritional anemia | 18 (30.0%) | 12 (27.3%) | 0.94 |

**Supplementary Table 2:** Region-stratified distribution of different causes of incident anemia among those with anemia in LASI-DAD

| **Anemia type** | **REGION1 (n=78, %)** | **REGION 2 (n=43, %)** | **REGION 3 (n= 49, %)** | **REGION 4**  **(n= 67, %)** | **p-value** |
| --- | --- | --- | --- | --- | --- |
| **Nutritional Anemia** | 50 (64.1%) | 25 (58.1%) | 23 (46.9%) | 33 (49.3%) | 0.15 |
| Isolated iron deficiency anemia | 26 (52.0%) | 16 (64.0%) | 11 (47.8%) | 13 (39.4%) | 0.40 |
| Isolated Vit B12 deficiency anemia | 8 (16.0%) | 3 (12.0%) | 3 (13.0%) | 3 (9.1%) | 0.79 |
| Isolated folate deficiency anemia | 4 (8.0%) | 1 (4.0%) | 2 (8.7%) | 8 (24.2%) | 0.05 |
| Multiple nutritional deficiency anemia | 12 (24.0%) | 5 (20.0%) | 7 (30.4%) | 9 (27.3%) | 0.98 |
|  |  |  |  |  |  |
| **Non-nutritional anemia** | 27 (34.6%) | 18 (41.9%) | 26 (53.1%) | 34 (50.7%) | 0.15 |
| Anemia of chronic disease/inflammation | 9 (33.3%) | 6 (33.3%) | 12 (46.2%) | 15 (44.1%) | 0.76 |
| Anemia of chronic kidney disease | 4 (14.8%) | 2 (11.1%) | 2 (7.7%) | 2 (5.9%) | 0.85 |
| Multiple non-nutritional causes anemia | 5 (18.5%) | 5 (27.8%) | 8 (30.8%) | 3 (8.8%) | 0.21 |
| Unexplained non-nutritional anemia | 9 (33.3%) | 5 (27.8%) | 4 (15.4%) | 14 (41.2%) | 0.34 |

NOTES:

Percentages are column percentages at a significance level of 0.05

* Regions:

Region 1: Jammu and Kashmir, Punjab, Uttaranchal, Haryana, Delhi, Rajasthan, Uttar Pradesh, Bihar.

Region 2: Maharashtra, Gujrat, and Madhya Pradesh.

Region 3: Andhra Pradesh, Karnataka, Kerala, Tamil Nadu, Pondicherry, Telangana.

Region 4: Assam, West Bengal, Jharkhand, Odisha, Chhattisgarh
